# Management and Outcomes of Diabetic Foot Complications Requiring Surgical Intervention at a Sudanese First‐Level Hospital: A Prospective Clinical Audit

**DOI:** 10.1002/hsr2.72278

**Published:** 2026-04-11

**Authors:** Ibrahim Abusufian Elkabashi Dafallah, Duha Elfatih Elamin Awadalla, Ahmed Fathalrahman Zainalabdin, Hammam Mohamed Awadelkareem Badawi, Mohammed Elfatih Elamin Awadalla, Nazik Mahmoud Abdelaal Abeldaifa, Ahmed Mohamed Awadelkareem Badawi

**Affiliations:** ^1^ Faculty of Medicine Alzaiem Alazhari University Khartoum North Sudan; ^2^ Faculty of Medicine Omdurman Islamic University Omdurman Sudan; ^3^ General Surgery, Faculty of Medicine University of Bahri Khartoum North Sudan; ^4^ Faculty of Medicine University of Science and Technology Khartoum Sudan; ^5^ Faculty of Medicine Neelain University Khartoum Sudan; ^6^ Department of Medicine Omdurman Military Hospital Omdurman Sudan; ^7^ Faculty of Medicine University of Khartoum Khartoum Sudan

**Keywords:** clinical audit, diabetes mellitus, diabetic foot ulcers, hygiene in surgical procedures, patient education, Sudan

## Abstract

**Background and Aims:**

Diabetic foot complications, encompassing ulcers, abscesses, and tissue necrosis, are common and serious complications of diabetes mellitus that can significantly impact quality of life and potentially lead to amputation, particularly in the presence of risk factors and inadequate care. This prospective clinical audit evaluated the management and outcomes of diabetic foot complications at a first‐level hospital in Hilaliya, Sudan.

**Methods:**

A prospective clinical audit of 15 patients requiring surgical intervention was conducted from August to November 2023. Data on demographics, ulcer characteristics, timeliness of care, hygiene in surgical procedures, antibiotic use, and patient education were collected and analyzed.

**Results:**

Patients had a median age of 54 years and interquartile range (IQR) of 40–65 years. Ulcers were reported in 46.7% of cases and abscesses in 20%. Multidisciplinary team assessments within 24 h were provided to 53.3% of patients. Timely surgical intervention was performed in 93.3% of patients. Hygiene in surgical procedures measures were documented in all cases and sterile field usage was recorded in 20%. Empirical antibiotics were prescribed to 93.3% of patients. Complete healing without complications occurred in 86.7% of cases and 13.3% underwent amputation. Regarding patient education, 46.7% of patients had limited awareness of foot care and 53.3% received no counseling.

**Conclusion:**

There are significant gaps in hygiene in surgical procedures (particularly sterile protocols) and patient education. Urgent institutional focus on interdisciplinary coordination, sterile technique enforcement, and structured education programs is needed to improve outcomes.

## 1 Introduction

Diabetic foot complications are common and serious challenges of diabetes mellitus. Individuals with diabetes face a lifetime risk of up to 34% of developing diabetic foot ulcers (DFUs) [1], which affect approximately 18.6 million people worldwide. DFUs are the most prevalent type of diabetic foot complications and often progress to diabetic foot infections (DFIs). DFIs are severe conditions characterized by tissue necrosis that significantly increases the risk of hospitalization and lower‐extremity amputation. Without timely and effective management, approximately 50% of DFUs may become infected [2].

The understanding of these complications has advanced significantly since the early 20th‐century with the term “diabetic gangrene.” They are now recognized as multifactorial conditions driven by neuropathy, peripheral arterial disease (PAD), and impaired immune responses [3]. Current practices rely heavily on clinical assessment, often utilizing various classification systems such as SINBAD, Wagner, and Texas classification systems to categorize ulcer severity, alongside microbiological investigations to identify causative pathogens [4, 5].

These conditions impose a significant burden on patients and healthcare systems through prolonged hospital stays, high costs, amputations, and reduced quality of life [6]. In Africa, the prevalence is approximately 13% and is increasing [7]. While amputation and mortality rates have decreased in some regions, they remain unacceptably high [4]. Studies from Nigeria, India, Saudi Arabia, and Ethiopia confirm these adverse outcomes, which are often linked to disparities in healthcare access, socioeconomic factors, and preventative care [8, 9, 10, 11, 12, 13, 14]. Diabetic foot complications often reflect systemic vascular and inflammatory pathology with markedly elevated risks of cardiovascular morbidity and mortality, cerebrovascular events, chronic kidney disease, and overall mortality, often requiring interdisciplinary management [15].

In Sudan, where the adult diabetes incidence was 13.7% in 2019 (affecting 2.5 million people), limited data exist on the burden of diabetic foot complications [16]. A previous clinical audit at a Sudanese hospital revealed that only 40% of patients with DFI avoided amputation, raising serious questions about the quality of care [5].

The pathogenesis of diabetic foot complications encompasses varying contributions of neuropathy, ischemia, and infection. For example, while neuropathic ulcers have historically been considered the most common type of DFU, research has indicated that ischemic ulcers often present with severe clinical features and worse outcomes [15]. PAD, a major contributing factor, has a substantial prevalence in patients with diabetic foot complications and it significantly impacts limb salvage [17]. Infections further complicate the clinical picture, with *Staphylococcus aureus* and *Pseudomonas aeruginosa* being frequently implicated as common causative microorganisms [18, 19].

Current management strategies for diabetic foot complications predominantly revolve around an interdisciplinary team approach [20], encompassing patient education, wound debridement, offloading techniques, advanced dressings, surgical interventions, and antimicrobial therapy [21]. The importance of early screening and regular foot assessments has been emphasized; however, in practice, there are frequently delays in referral to specialized care, which negatively impact outcomes [22, 23].

The International Working Group on the Diabetic Foot (IWGDF) Guidelines 2023 recommend an interdisciplinary team approach to diabetic foot care, emphasizing early diagnosis, appropriate empiric antibiotic therapy, and effective wound management. Key recommendations include surgical intervention when necessary, offloading techniques to alleviate pressure on ulcers and comprehensive patient education on complication care and infection recognition [6, 24].

Given the pressing need to improve diabetic foot complication care in Sudan, particularly in light of the patient burden and outcomes shown in previous studies and via a preliminary internal review showing inconsistencies in diabetic foot care, this prospective clinical audit aimed to evaluate the management of diabetic foot complications at Hilaliya first‐level hospital to enhance quality and safety. We assessed adherence to established guidelines and analyzed patient outcomes. This study is the sole audit of diabetic foot care in Sudanese primary hospitals which provides important initial benchmarks for improving diabetic foot care in low‐resource settings.

## 2 Methods

### 2.1 Study Design

This study was conducted as a prospective clinical audit. Its primary purpose was to systematically assess and improve local care against IWGDF guidelines. The IWGDF guidelines address diabetic foot complications, a term that encompasses the full spectrum of diabetic foot complications as defined in this study. Data were collected via a prospective design to evaluate care provided to patients with diabetic foot complications requiring surgical intervention.

For the purposes of this study, the following definitions are used throughout the manuscript to ensure a standardized approach:

Diabetic foot complications: A diabetic foot complication represents any pathological alteration of pedal skin integrity or underlying soft tissues in individuals with diabetes mellitus, resulting from the complex interplay of neuropathy, ischemia, and mechanical stress.

Diabetic foot infection: A patient with diabetes presenting with a foot ulcer exhibiting at least two of the following characteristics: local swelling or induration, erythema > 0.5 cm beyond the ulcer edge, tenderness or pain, warmth compared with the surrounding area or purulent discharge (pus) from the ulcer.

Multidisciplinary team (MDT): A collaborative group of healthcare providers from at least three distinct roles, including a physician (e.g., general practitioner or internist), a surgeon, and a nurse responsible for diabetic foot complication care. The MDT must jointly assess the patient and document a coordinated care plan [6].

Hygiene in surgical procedures: Full compliance requires documentation of all four hygiene in surgical procedures (hand hygiene, sterile instruments, waste disposal, sterile field) for a single patient encounter.

Deformities: Acquired structural abnormalities of the foot and ankle resulting from the long‐term sequelae of diabetes including Charcot neuroarthropathy, digital deformities, prominent metatarsal heads, and collapse of the longitudinal arch or other joint dislocations/subluxations.

### 2.2 Participants and Setting

Data were collected between August 2023 and November 2023 at Hilaliya Hospital, a first‐level facility serving approximately 15,000 people from Hilaliya and surrounding towns with an average of 700 patient visits monthly. The hospital consists of an emergency department, general physician clinic and one to two outpatient clinics daily. The hospital capabilities include basic lab investigations, ultrasound, electrocardiography tracing and major and minor operating theaters. The inclusion criteria were as follows: (1) adult patients (≥ 18 years of age) with a diagnosis of diabetes mellitus (Type 1 or Type 2); (2) patients who presented to the hospital with a foot complication related to their diabetes; and (3) patients who needed surgical intervention as determined by the treating physician. The exclusion criteria were as follows: (1) patients who presented with diabetic foot complications but did not require surgical intervention during the data collection period; and (2) patients who declined to participate in the study after informed consent was obtained. All eligible patients during the study period were included. The final sample size was 15 participants, determined by the number of eligible patient visits during the specified time frame.

### 2.3 Instruments and Measurement

The primary data collection instrument was a structured questionnaire containing quantitative elements to capture specific data points (e.g., age, time to intervention, and antibiotic use). Four trained investigators collected data via a structured English questionnaire developed through a literature review and expert consultation. While the questionnaire was developed on the basis of a literature review and expert opinion to ensure content validity, formal validity and reliability testing were not conducted prior to the study. The variables included demographics, diabetes history, complication characteristics, timeliness of assessment (< 24 h) and intervention, MDT involvement, antibiotic use, hygiene in surgical procedures compliance, surgical details, patient education, and 3‐month outcomes (complications, amputation). New‐onset neuropathy or deformities were recorded only if documented as absent at baseline and diagnosed during follow‐up.

The audit goals derived from IWGDF guidelines were as follows: (1) ≥ 90% of patients receive MDT assessment within 24 h; (2) ≥ 60% undergo weekly MDT follow‐up; (3) ≥ 90% demonstrate full hygiene in surgical procedures compliance; (4) ≥ 90% receive timely surgical intervention; (5) ≥ 80% receive appropriate dressing. Appropriate dressing is defined based on infection severity per IWGDF/IDSA criteria: moisture‐retentive dressings for uninfected/mild infections (IWGDF 1), antimicrobial dressings (silver or polyhexamethylene biguanide) for moderate infections (IWGDF 2), and saline‐moistened gauze packing following surgical debridement for severe infections (IWGDF 3); (6) ≥ 60% compliance with antibiotic stewardship principles (appropriate selection on the basis of published RCT evidence, monitoring, and duration); and (7) ≥ 80% receive patient education. The thresholds were set through expert consultation to reflect contextually appropriate and achievable goals.

### 2.4 Data Collection

Prospective observational data were collected via the structured online questionnaire. The questionnaire included the following variables: patient demographics (age, sex), date of presentation, time since diabetes diagnosis, current diabetes treatment regimen, foot complication characteristics (type, location), evaluation within 24 h of presentation (yes/no), receipt of MDT follow‐up (yes/no), operating theater status during treatment, timeliness of intervention (time from presentation to intervention), and patient outcomes (e.g., complications, need for further intervention, amputation). Data were also collected on antibiotic use and knowledge regarding prevention of diabetic foot complications such as use of appropriate footwear/pressure reduction, improving perfusion, glycemic control, foot checking/self monitoring, and recognizing early signs of infection. The questionnaire also assessed the provision of patient education regarding diabetes management and lesion care. Patients were followed up for 3 months for complications or changes in treatment. The questionnaire used is fully shared in the Supporting Files.

### 2.5 Data Management and Statistical Analysis

The data were securely stored in Google Sheets and analyzed via IBM SPSS Statistics (v28.0.0). The Kolmogorov–Smirnov test was used to assess the normality of continuous variables. Normally distributed data are presented as mean ± standard deviation; non‐normally distributed data are presented as median and interquartile range (IQR). Categorical data are summarized as frequencies and percentages. Compliance with audit standards was evaluated descriptively through direct comparison of observed proportions to goal targets.

### 2.6 Ethical Approval and Consent to Participate

In line with guidelines from the Committee on Publication Ethics (COPE) [25], this study was classified as a prospective clinical audit. Approval was granted by the hospital administration on the basis that the study design protected patient welfare and ensured data anonymity.

Informed consent was obtained from all participants prior to data collection. All patient data were anonymized by assigning a participant ID at the source to ensure confidentiality. The audit was conducted in accordance with the principles of the Declaration of Helsinki [26]. This manuscript was prepared following the SQUIRE 2.0 guidelines for reporting quality improvement studies and the SAMPL guidelines for reporting statistical analyses [27, 28].

## 3 Results

### 3.1 Patient Characteristics and Foot Complication Presentation

**Figure 1:**
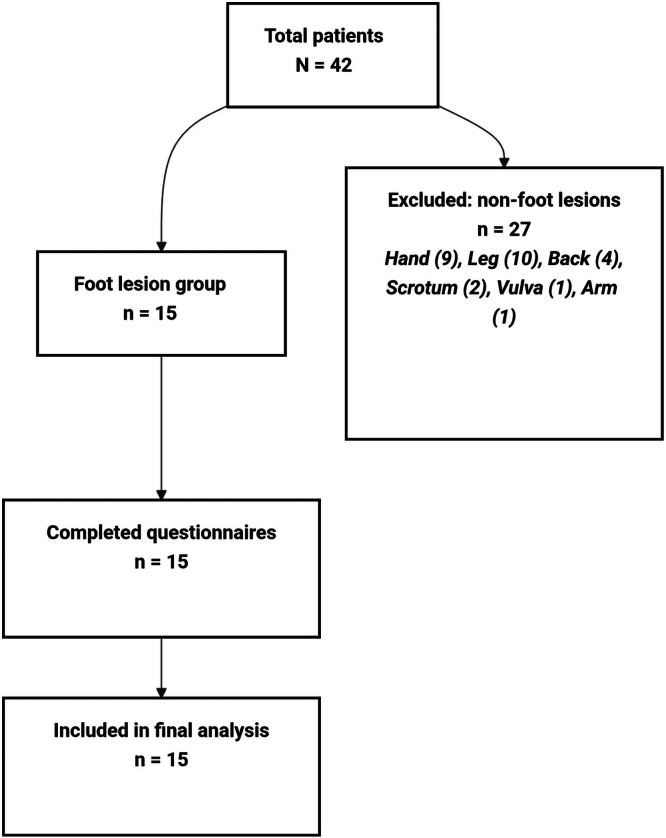
Participant flow diagram.

Of the 42 patients assessed, 27 were excluded due to non‐foot complications. The remaining 15 patients with foot complications all completed the questionnaires and were included in the analysis (Figure 1). The study group consisted of 15 patients with a median age of 54 years (IQR: 40–65 years). None of the patients declined participation. Fourteen patients (93.3%) were residents of Hilaliya, whereas one patient (6.7%) lived in Baranku. The most common complication types observed were ulcer, affecting seven patients (46.7%), and abscesses, which were identified in three patients (20%). The less frequent presentations included cellulitis, wet gangrene, and necrotizing fasciitis which were each diagnosed in one patient (6.7%). One patient (6.7%) presented with dry gangrene secondary to chronic ischemia with a pre‐existing foot ulcer. Additionally, one patient (6.7%) presented with an ingrown nail.

### 3.2 Assessment and Intervention Timeliness

An MDT assessment within 24 h was conducted for 8 (53.3%) of the 15 total patients. A third of patients with abscesses, one out of three (33.3%) received this assessment, whereas four out of seven (57.1%) patients with an ulcer received the assessment. All three patients suffering from cellulitis, necrotizing fasciitis, and ingrown nail complication types, respectively, received MDT assessment. A greater percentage of patients (53.3%) received an MDT assessment within 24 h than did those who did not (46.7%).

Timely intervention within 24 h was implemented for 14 (93.3%) of the 15 total patients. All patients with abscesses, cellulitis, wet gangrene, dry gangrene, necrotizing fasciitis, and ingrown nail complication types received timely intervention. Only one case of an ulcer did not receive timely intervention (14.3%). 93.3% received timely intervention within 24 h, whereas only 6.7% did not.

### 3.3 Hygiene in Surgical Procedures and Antibiotic Use

The implementation of various hygiene in surgical procedures measures was assessed across all cases. While any measure was documented in all 15 cases (100%), full compliance with all 4 measures occurred in only 2 cases (13.3%). Specifically, hand hygiene measures were documented in 10 cases (66.7%), whereas the use of aseptic tools and techniques and appropriate disposal of contaminated materials were both documented in 12 cases (80%). The least common documented measure was the use of a sterile field, which was recorded in only three cases (20%).

**Figure 2:**
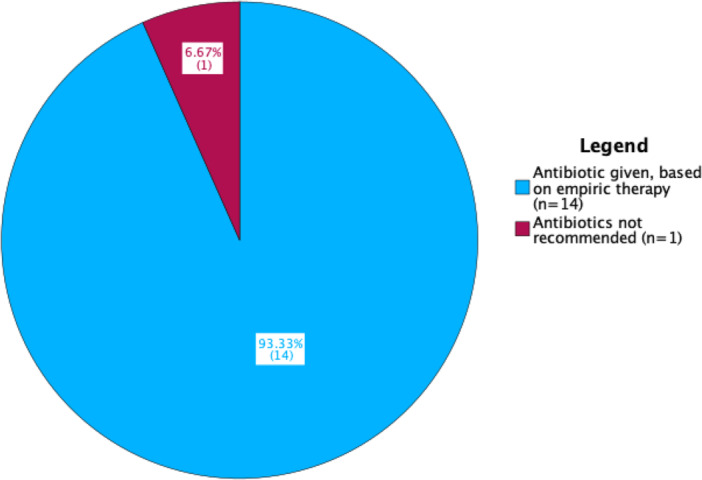
Antibiotic prescription for diabetic foot complications.

Fourteen patients (93.3%) received empirical antibiotics. Empirical antibiotics consisted of amoxicillin/clavulanate with or without metronidazole for anaerobic bacteria coverage. Of the 14, 13 (86.7%) presented with clinical signs of infection (e.g., purulence, erythema). The one patient that did not present with clinical signs of infection presented with dry gangrene. In this case, empirical antibiotics were administered to prevent infection despite no clinical signs of infection. The one patient (6.7%) that did not receive antibiotic was a DFU where antibiotic was not indicated by the attending physician (Figure 2).

### 3.4 Foot Complication Outcomes

The outcomes showed that 12 ulcers (80%) completely healed without complications and 2 required amputation (*n* = 2, 13.3%).

During the follow‐up duration, one patient was diagnosed with neuropathy (*n* = 1, 6.7%) and one was diagnosed with Charcot deformity (*n* = 1, 6.7%).

### 3.5 Patient Education

Patient awareness regarding diabetic complication care was assessed and patients were categorized into three grades. Two patients (13.3%) demonstrated high awareness, with knowledge of four to five prevention points. Six patients (40%) reported either average awareness (two to three prevention points) and seven (46.7%) had low awareness (one or fewer prevention points) regarding diabetic foot complications.

The timing of post presentation counseling was also recorded. Counseling about diabetic complication care was provided within the first week of presentation for six patients (40%). Only one patient (6.7%) received counseling after the first week. However, eight patients (53.3%) did not receive any follow‐up counseling (*n* = 8, 53.3%).

Adherence to established audit goals was evaluated. A thorough assessment by an MDT within 24 h of presentation was achieved in 53.3% of the cases. Only 46.7% of patients underwent a weekly follow‐up evaluation by an MDT. Compliance with full hygiene in surgical procedures measures was achieved in only 13.3% of the cases.

Effective surgical intervention within the recommended time frame was achieved in 93.3% of the cases. Appropriate wound dressing on the basis of the severity of DFI was also achieved in 93.3% of the patients. Furthermore, the initiation of empirical antibiotics was achieved in 93.3% of the cases. The provision of patient education and diabetic foot complication care instructions was achieved in 46.7% of the cases.

## 4 Discussion

This study found that there is room for improvement in the care of individuals with DFUs at Hilaliya Hospital. Our study aligns with the literature regarding the peak incidence of diabetic foot complications in the fifth and sixth decades of life [7, 18]. The observed predominance of abscesses and ulcers is consistent with the varied clinical presentations of diabetic foot complications, which include infected and noninfected complications [29, 30]. Our study revealed a predominance of ulcers and abscesses which are more likely to arise in diabetics due to a combination of neuropathy, trauma, and vascular insufficiency [6]. Some patients present with cellulitis and necrotizing fasciitis, which require timely intervention, as these conditions are associated with increased morbidity and risk of limb loss if not addressed promptly [31].

In our study, only 53.3% of patients met the standard for a thorough MDT assessment within 24 h. Notably, while MDT assessments may not have been conducted in these cases, surgeons may decide to proceed to operate immediately given the urgent nature of some diabetic foot complications. Current approaches for the assessment and timely intervention of diabetic foot complications are largely guided by the urgency of limb‐threatening infections and the risk of rapid progression [32]. The established gold standard underscores the importance of a prompt and comprehensive evaluation by an MDT within 24 h of a patient's presentation [33].

Despite the clear benefits of timely intervention, several challenges limit its effectiveness. Patient‐related factors, such as delays in seeking care due to poor health literacy, denial, or fear, contribute significantly to delayed presentations [21]. Socioeconomic factors, including a lack of access to specialized care, geographical barriers, and inadequate insurance coverage, often exacerbate these delays [7]. From a healthcare provider perspective, a lack of standardized pathways for referrals, challenges in coordinating care among different specialties, and limited resources, such as staffing shortages and the unavailability of diagnostic tools, can also hinder timely assessment [34]. In our study, there were differences between assessment and intervention timeliness in the abscess and ulcer categories, where the MDT assessment rates were the lowest (33.3% and 57.1%, respectively), yet timely interventions were achieved for all patients with abscesses and 85.7% of ulcer patients. This could be due to the assumed benign nature of DFUs compared with other complications, such as gangrene and necrotizing fasciitis.

In our study, while documentation of at least one hygiene measure occurred in all cases, the specific implementation of hand hygiene (66.7%) and the use of a sterile field (20%) were notably lower than the use of aseptic tools and techniques and appropriate disposal of contaminated materials (both 80%). Empirical antibiotic therapy was employed in the vast majority of patients (93.3%) because of the absence of laboratory culture and sensitivity data, due to resource and logistical constraints. In the case of dry gangrene in our study, antibiotics targeted infected ulcer components, not necrosis itself. In our audit, full compliance with hygiene in surgical procedures was found in only 13.3% of cases, significantly below the 90% goal.

The current approaches to hygiene in surgical procedures in diabetic foot lesion management include hand hygiene, aseptic techniques, and proper disposal of contaminated materials [18, 35]. While the majority of cases reported adequate aseptic techniques and disposal, the lower compliance with hand hygiene highlighted potential issues with staff training. The sterile field, which was even less consistently used, highlights resource constraints common in developing countries that limit the translation of theoretical knowledge into best practice implementation. These resource limitations also extend to diagnostic capacity, where the absence of laboratory culture and sensitivity testing necessitates reliance on empirical antibiotic usage [35].

Many patients presenting with diabetic foot complications had low awareness of their condition; this was coupled with a lack of follow‐up counseling and education mainly due to poor planning of patient counseling duties between surgeons and primary care providers. This low awareness finding is similar to recent research in Nigeria [7]. Moreover, studies have demonstrated that patients from lower socioeconomic backgrounds are at greater risk of developing diabetic foot complications [36]. Furthermore, global epidemiological studies show regional variations, with higher rates in certain geographic areas. The prevalence of DFUs in Saudi Arabia, for example, is more than twice as high as that in Iraq [15].

In our study, the high rates of timely surgical intervention (93.3%) and wound dressing compliance (93.3%) were not reflected by high rates of appropriate patient education and understanding. This highlights the focus on acute and technical care with less focus on preventative care and education. This finding is widespread among various health centers and many patients presenting upon referral or after treatment have poor knowledge about preventing diabetic foot complications [21]. Only 13.3% of patients demonstrated high awareness of diabetic foot complications. This low health literacy in the population we treat represents a significant area for improvement.

Data from a national diabetic septic foot audit in the United Kingdom indicated that 49% of foot complications healed within 3 months, highlighting the impact of advancements in surgical and nursing techniques [37, 38]. Our own experience reflects these positive trends and demonstrates that we are already on the right track, as only two patients (13.3%) required amputation in the acute setting, a notable improvement compared with a prior Sudanese audit where amputation was avoided in only 40% of patients [4]. This outcome aligns well with a systematic review of 16 studies, suggesting that the right interventions can enhance recovery and reduce complications [39].

Current approaches to patient education play a vital role in managing diabetes, emphasizing key areas such as foot care, blood glucose control, and medication adherence [20, 40]. Research highlights that educational interventions and screening programs can significantly contribute to preventing diabetic foot complications [6, 41]. While traditional didactic methods provide essential information, there are opportunities to enhance these approaches by incorporating individual needs, cultural contexts, and varying health literacy levels to support sustainable diabetic foot complication management [42].

### 4.1 Limitations

The tracking of outcomes has several limitations. First, there was significant variation in the care providers and hospitals that patients visited for debridement which could have affected our data. Additionally, it was challenging to ascertain patients' previous visits to the hospital, leading to gaps in patient history. Another limitation was the lack of documentation regarding the properties of diabetic foot complications both before and after referrals to ascertain which complications may have occurred owing to treatment at a different institution. Furthermore, patients with a possible history of diabetic foot complications were treated as first‐visit patients because of the constraints of the defined data collection period. The relatively short median follow‐up period of 3 months, limited our ability to assess long‐term complications. The MDT composition (physician, surgeon, and nurse) reflects local staffing constraints and differs from the IWGDF recommendations for specialized teams (e.g., podiatrist and diabetes nurse specialist), potentially limiting comprehensive assessments. The small sample size limits the statistical power but provides pilot data for resource‐limited settings. The use of a context specific yet unvalidated questionnaires may affect assessment reliability. Details regarding the specific offloading device, duration of use, and adherence were not consistently documented. While patient education on offloading was recorded, the absence of this information limits our ability to fully contextualize treatment outcomes. Finally, the observational nature of this audit means our findings are susceptible to performance bias as clinicians were aware their practice was being evaluated, which may have influenced their behavior.

## 5 Conclusion

In conclusion, this prospective clinical audit shows considerable room for improvement in the management of diabetic foot complications at our hospital. Important gaps were identified in adherence to IWGDF standards, particularly in timely MDT assessment, surgical hygiene practices, and patient education, while high compliance was observed in surgical intervention, wound care, and empirical antibiotic use. These findings highlight the need for stronger institutional systems, including rapid referral pathways, standardized documentation, patient education, and regular staff training in evidence‐based diabetic foot care. Further re‐audit is recommended to evaluate the impact of these quality improvement measures on guideline adherence and patient outcomes.

## Author Contributions

**Ibrahim Abusufian Elkabashi Dafallah:** conceptualization, methodology, validation, project administration, data curation, writing – original draft. **Duha Elfatih Elamin Awadalla:** methodology, investigation, software, data curation, writing – original draft, visualization. **Ahmed Fathalrahman Zainalabdin:** conceptualization, methodology, resources, supervision. **Hammam Mohamed Awadelkareem Badawi:** data curation, investigation, methodology. **Mohammed Elfatih Elamin Awadalla:** software, formal analysis. **Nazik Mahmoud Abdelaal Abeldaifa:** software, investigation, supervision. **Ahmed Mohamed Awadelkareem Badawi:** writing – review and editing. All authors have read and approved the final version of the manuscript.

## Funding

The authors have nothing to report.

## Disclosure

The lead author Ibrahim Abusufian Elkabashi Dafallah affirms that this manuscript is an honest, accurate, and transparent account of the study being reported; that no important aspects of the study have been omitted; and that any discrepancies from the study as planned (and, if relevant, registered) have been explained.

## Ethics Statement

The authors received approval to conduct the study from the Institutional Review Board and Administration at Hilaliya Hospital. The study adhered to ethical standards and guidelines. Informed consent was obtained from all participants. The audit adhered to strict patient confidentiality and data protection guidelines. The data were anonymized and stripped of any identifying information to maintain patient privacy and compliance with ethical standards. The authors confirm that all methods were conducted in accordance with relevant research ethics guidelines and regulations.

## Consent

Written informed consent for the publication of anonymized data was obtained from all participants. This included consent to publish aggregated findings without any personally identifiable information.

## Conflicts of Interest

The authors declare no conflicts of interest.

## Supporting information


Supporting File

## Data Availability

Ibrahim Abusufian Elkabashi Dafallah had full access to all of the data in this study and takes complete responsibility for the integrity of the data and the accuracy of the data analysis. The data sets used and analyzed during the current study are available and can be accessed through the following link: https://osf.io/h4j5r.
