# Supplementary material for: Management and Outcomes of Diabetic Foot Complications Requiring Surgical Intervention at a Sudanese First‐Level Hospital: A Prospective Clinical Audit
Source: Health Sci Rep. 2026 Apr 11;9(4):e72278. doi: 10.1002/hsr2.72278 (PMC13069585; doi:10.1002/hsr2.72278)
Supplement: Supplementary file 1 — Supporting File [file HSR2-9-e72278-s001.pdf]

Clinical Audit

Description

* Indicates required question

1. Patient ID *

2. Age

3. Date of Presentation *

Example: January 7, 2019

4. Residence (Town)

Mark only one oval.

- Hilaliya
- Abu Ushar
- Other:

5. Patient Hx

Check all that apply.

- [ ] Callus
- [ ] Smoker
- [ ] HTN
- [ ] Visual complications
- [ ] Use of appropriate footwear
- [ ] Renal insufficiency/ CKD
- [ ] Other: ____________________

6. Time since Diabetes Diagnosis

____

7. Current diabetes treatment

Check all that apply.

- [ ] Insulin
- [ ] Metformin
- [ ] Vilget (Vildagliptin)
- [ ] Pamilintide
- [ ] Canagliflozin
- [ ] Glimepride (Amaryl)
- [ ] None
- [ ] Other: ____________________

8. Patient education/awareness *

Check all that apply.

- [ ] Use of appropriate footwear/pressure reduction
- [ ] Blood perfusion
- [ ] Glycemic control
- [ ] Foot checking/Self monitoring
- [ ] Recognizes early signs of infection
- [ ] Patient is not aware about diabetic septic ulcers

9. Type of diabetic ulcer *

Check all that apply.

- [ ] Wound
- [ ] Abscess
- [ ] Cellulitis
- [ ] Dry Gangrene
- [ ] Wet gangrene
- [ ] Necrotizing fasciitis
- [ ] Other: ____________________

10. Location of ulcer (IF ON FOOT)

Mark only one oval per row.

	Right	Left
**Dorsal foot**	⬭	⬭
**Hind foot**	⬭	⬭

11. Location of ulcer (BODY)

Mark only one oval.

- Hand
- Arm
- Axilla
- Knee
- Calf
- Thigh
- Back
- Other: ____

12. Was pt. assessed by a within 24 hours of presentation? *

Mark only one oval.

- Yes, the pt. was assessed by at least 2 members of multidisciplinary team
- Yes, the pt. was assessed by hospital staff
- No
- Other: ____

13. Did patient undergo multidisciplinary follow up evaluation (with regards to counseling, investigations and diabetes management etc.) during the week? *

Mark only one oval.

- ( ) Yes, patient underwent follow up evaluation
- ( ) Yes, patient underwent partial follow up evaluation
- ( ) No, due to pt. compliance
- ( ) No,
- ( ) Not a follow up (first presentation)
- ( ) Other:

14. Status of operating room throughout diabetic septic ulcer treatment *

Check all that apply.

- [ ] Handwashing/Clean hands/Gloves
- [ ] Use of aseptic tools & techniques
- [ ] Decontamination zone
- [ ] Appropriate disposal of contaminated materials
- [ ] No intervention conducted

15. Did pt. undergo effective and timely intervention within the appropriate timeframe? *

Check all that apply.

- [ ] Dressing (within 24 hrs)
- [ ] Debridement (with 24 hrs)
- [ ] Abscess drainage (within 3 days)
- [ ] Amputation (within 3 days)
- [ ] No intervention was conducted/failed
- [ ] No intervention recommended

16. Outcomes *

Check all that apply.

- [ ] Patient 1st Visit
- [ ] Patient follow up visit
- [ ] Foot amputation
- [ ] Amputation
- [ ] Deformity
- [ ] Non-healing
- [ ] Complicated ulcer
- [ ] Neuropathy
- [ ] Referral to another hospital
- [ ] Fully healed without complication

17. Use of antibiotic *

Mark only one oval.

- ( ) Not recommended
- ( ) Given, based on empiric therapy
- ( ) Given, based on culture sensitivity
- ( ) No antibiotic was given

18. Patient education of diabetic septic ulcers *

Mark only one oval.

- ( ) Patient received counseling within 1st week of presentation
- ( ) Patient received counselling after 1st week of presentation
- ( ) Patient did not receive counselling before
